# Severely low serum magnesium is associated with increased risks of positive anti-thyroglobulin antibody and hypothyroidism: A cross-sectional study

**DOI:** 10.1038/s41598-018-28362-5

**Published:** 2018-07-02

**Authors:** Kunling Wang, Hongyan Wei, Wanqi Zhang, Zhen Li, Li Ding, Tong Yu, Long Tan, Yaxin Liu, Tong Liu, Hao Wang, Yuxin Fan, Peng Zhang, Zhongyan Shan, Mei Zhu

**Affiliations:** 1 Department of Endocrinology and Metabolism, Tianjin Medical University General Hospital, No154 Anshan Road, Heping District, Tianjin, 300052 China; 2 Department of Nutrition and Food Hygiene, School of Public Health, Tianjin Medical University, No22 Qixiangtai Road, Heping District, Tianjin, 300070 China; 3 Department of Nuclear Medicine, Zhujiang Hospital of Southern Medical University, No 253 Gongye Road, Guangzhou, Guangdong Province 510282 China; 4 Department of Endocrinology, Huaihe Hospital of Henan University, Kaifeng, No 8 Baobei Road, Henan Province 475000 China; 5 Department of Endocrinology and Metabolism and Institute of Endocrinology, The First Hospital of China Medical University, Shenyang, Liaoning Province 110001 China

## Abstract

Trace elements, such as iodine and selenium, are closely related to autoimmune thyroiditis and thyroid function. Low serum magnesium is associated with several chronic diseases; however, its associations with autoimmune thyroiditis and thyroid function are unclear. We investigated the relationships between low serum magnesium, autoimmune thyroiditis, and thyroid function in 1,257 Chinese participants. Demographic data were collected via questionnaires, and levels of serum thyroid stimulating hormone, anti-thyroid peroxidase antibody, anti-thyroglobulin antibody (TGAb), free thyroxine, serum magnesium, serum iodine, and urinary iodine concentration were measured. Participants were divided into serum magnesium level quartiles (≤0.55, 0.551–0.85, 0.851–1.15, and >1.15 mmol/L). The median serum magnesium level was 0.89 (0.73–1.06) mmol/L; levels ≤0.55 mmol/L were considered severely low (5.9% of participants). The risks of TGAb positivity and Hashimoto thyroiditis (HT) diagnosed using ultrasonography in the lowest quartile group were higher than those in the adequate magnesium group (0.851–1.15 mmol/L) (p < 0.01, odds ratios [ORs] = 2.748–3.236). The risks of total and subclinical-only hypothyroidism in the lowest quartile group were higher than those in the adequate magnesium group (0.851–1.15 mmol/L) (p < 0.01, ORs = 4.482–4.971). Severely low serum magnesium levels are associated with an increased rate of TGAb positivity, HT, and hypothyroidism.

## Introduction

Magnesium is the fourth most abundant essential mineral in the human body after sodium, potassium, and calcium^1^, and is a cofactor for more than 300 enzymes that regulate a variety of biochemical processes, such as DNA/RNA synthesis, protein synthesis, oxidative phosphorylation, and glycolysis^1,2^. Magnesium is mainly absorbed through the diet, and high-magnesium foods include nuts, seeds, whole grains, and leafy greens. Epidemiological surveys show that magnesium deficiency exists in many regions worldwide^3–5^. According to data from the National Health and Nutrition Examination (2001–2010) in the United States, the magnesium intakes of only 18.8% of male participants and 24.8% of female participants met the recommended dietary allowance^3^. The Nutrition and Health Survey in Taiwan (NAHSIT) also showed that the daily intakes of magnesium in 75% of male participants and 81% of female participants were lower than the dietary reference intakes (DRIs)^4^. It is estimated that the prevalence of low serum magnesium in the population is 2.5–15%^5^. Insufficient magnesium intake and low serum magnesium are associated with a variety of chronic diseases, including insulin resistance and type 2 diabetes mellitus^6,7^, metabolic syndrome^3,8^, hypertension^9^, cardiovascular disease^10^, stroke^11^, migraine^12^, attention deficit disorder^13^, Alzheimer’s disease^14^, and asthma^15^.

Magnesium is closely related to the immune system; *in vitro* experiments have showed that intracellular free magnesium ions are an important second messenger in the immune activation of T lymphocytes^16^ and B lymphocytes^17^, and magnesium channels and transport proteins play an important role in normal immune function^16,18,19^. Moreover, magnesium is also associated with cellular oxidative stress and inflammatory reactions^20^. The homeostasis of magnesium ions in mitochondria is important for cellular energy metabolism and for the ability to respond to oxidative stress^21^. The synthesis of glutathione, which is an important cellular antioxidant, is an ATP-dependent reaction and is therefore critically dependent on magnesium^1^. Studies have found that magnesium intake was inversely associated with levels of C-reactive protein, interleukin-6, and other inflammatory factors^6,22^, and that magnesium citrate supplementation can downregulate genes related to metabolic and inflammatory pathways^23^.

Autoimmune thyroiditis is a common endocrine disorder that is caused by a variety of environmental factors and is based on genetic susceptibility. A range of trace elements are related to the pathogenesis of autoimmune thyroiditis, among which the most important is iodine, followed by iron, selenium, and others^24,25^. There are few studies on the relationship between magnesium and thyroid disease. For example, a study on patients with Graves’ disease found that they exhibited a lower serum magnesium concentration than normal control participants, and that the serum magnesium concentration was negatively correlated with lymphocyte activation^26^. An Austrian study found that low serum magnesium was associated with abnormal thyroid function, which was improved after supplemental magnesium therapy^27^. To further clarify the relationship between serum magnesium levels and autoimmune thyroiditis, as well as thyroid function, we performed a cross-sectional study among the permanent residents of Tianjin.

## Results

### Demographic data of participants in different serum magnesium level groups

The demographic data of the study’s participants are shown in Table 1. A total of 1,257 participants were included, among whom the median serum magnesium level was 0.89 (0.73–1.06) mmol/L. The mean age of the participants was 42.5 ± 15.2 years and 49.2% were male. The proportion of elderly participants in the serum magnesium concentration ≤0.55 mmol/L quartile group was significantly higher than in the other groups. The 0.551–0.85 mmol/L quartile group had the lowest proportion of female participants. In terms of education and income levels, serum magnesium levels tended to increase gradually with increasing education and income levels (p < 0.0001). The proportion of non-smokers was lowest in the 0.551–0.85 mmol/L quartile group and increased gradually in the higher and lower quartile groups (p = 0.004). There were no significant differences in the proportions of body mass index (BMI) values among the groups.

**Table 1:** Characteristics of participants according to quartiles of serum magnesium.

Variables	All participants	Serum magnesium (mmol/L)				*p* value
		Quartile 1 ≤ 0.55	Quartile 2 0.551–0.85	Quartile 3 0.851–1.15	Quartile 4 > 1.15	
No. of participants (%)	1257 (100)	74 (5.9)	493 (39.2)	492 (39.1)	198 (15.8)	
Median of serum magnesium (quartile range, mmol/L)	0.89 (0.73–1.06)	0.50 (0.47–0.52)	0.73 (0.67–0.79)	0.98 (0.91–1.05)	1.26 (1.19–1.39)	
Age (years)	42.5 ± 15.2	46.0 ± 20.1	39.0 ± 14.6	43.5 ± 14.7	47.7 ± 13.4	0.000
Age group, %						
Young (18–39 years)	48.0	48.6	58.8	43.5	31.8	0.000
Middle-aged (40–64 years)	42.5	28.4	33.7	47.2	58.6	
Elderly (≥65 years)	9.5	23.0	7.5	9.3	9.6	
Sex (%)						
Male	49.2	44.6	60.9	56.1	33.8	0.000
Female	50.8	55.4	39.1	43.9	66.2	
Income (×1000 RMB/year, %)						
<10	4.5	14.9	4.5	4.3	1.0	0.000
10–50	49.6	56.8	54.2	47.0	42.4	
50–100	32.0	23.0	28.6	32.5	42.4	
≥100	13.9	5.4	12.8	16.3	14.1	
Smoking status (%)						
Never	71.0	75.7	64.2	74.6	77.3	0.004
Occasionally	1.5	0.0	1.8	1.4	1.0	
Frequently	27.5	24.3	33.9	24.0	21.7	
BMI (kg/m^2^)	24.7 ± 3.7	25.0 ± 4.6	24.4 ± 3.7	24.7 ± 3.7	25.1 ± 3.6	0.183
BMI (kg/m^2^) constitution, %						
<18.5 (marasmus)	3.3	4.1	3.2	3.3	3.5	0.379
18.5–23.9 (moderate)	42.7	44.6	44.8	41.1	40.5	
24–26.9 (overweight)	28.9	21.6	29.6	30.7	24.7	
≥27 (obese)	25.1	29.7	22.3	25.0	31.3	
Education						
Junior school or below	18.1	32.4	18.7	18.5	10.6	0.000
High school	54.2	56.8	58.6	50.4	52.0	
Junior college or a bove	27.7	10.8	22.7	31.1	37.4	

BMI: body mass index.

### Iodine nutrition state

The median urinary iodine concentration (UIC) of the subjects was 148.0 (quartile range, 89.9–227.4) μg/L, indicating that the iodine-related nutritional status of the population was at an appropriate level.

### Serum magnesium levels in the euthyroidism and hypothyroidism groups

The median serum magnesium level in the euthyroidism group was 0.88 (0.73–1.06) mmol/L, and that in the hypothyroidism group (including subclinical hypothyroidism) was 0.87 (0.61–1.09) mmol/L; there was no significant difference between the two groups (Z = −1.712, p = 0.087). The median serum magnesium level in the subclinical hypothyroidism group was 0.89 (0.60–1.10) mmol/L, which was not significantly different from that in the euthyroidism group (Z = −1.289, p = 0.197). The median serum magnesium level in the TPOAb-negative group was 0.88 (0.73–1.05) mmol/L, while that in the TPOAb-positive group was 0.90 (0.66–1.09) mmol/L; again, there was no significant difference between the two groups (Z = −0.663, p = 0.507). The median serum magnesium level in the TGAb-negative group was 0.88 (0.73–1.05) mmol/L, and that in the TGAb positive group was 0.91 (0.67–1.11) mmol/L, with no significant difference (Z = −0.221, p = 0.825).

### Relationship between serum magnesium level and thyroid disorders

The TPOAb positivity rate in the lowest serum magnesium level (≤0.55 mmol/L) quartile group was 29.7%, which was significantly higher than the rates in the other groups (χ^2^ = 10.703, p = 0.013). Moreover, the TGAb positivity rate in the lowest quartile group was 28.4%, which was also significantly higher than the rates in the other quartile groups (χ^2^ = 23.148, p = 0.000) (Table 2). The prevalence of Hashimoto thyroiditis (HT) diagnosed using ultrasonography in the lowest quartile group was 27.0%, which was significantly higher than in the other quartile groups (χ^2^ = 21.785, p = 0.000) (Table 2). The prevalence of subclinical hypothyroidism in the lowest quartile group was 32.4%, which was significantly higher than in the other quartile groups (χ^2^ = 40.490, p = 0.000); the prevalence of hypothyroidism overall (both clinical and subclinical) in the lowest quartile was 40.5%, which was also significantly higher than in the other groups (χ^2^ = 54.527, p = 0.000) (Table 2). The proportion of patients with clinical and subclinical hyperthyroidism was highest in the quartile 3 (0.851–1.15 mmol/L), while there were no patients with hyperthyroidism in the lowest quartile group. However, statistical analysis of patients with hyperthyroidism was not possible owing to their small number.

**Table 2:** Prevalence of thyroid disorders according to quartiles of serum magnesium.

	All (%)	Serum magnesium (mmol/L)				χ^2^	*p* value
		Quartile 1 ≤ 0.55	Quartile 2 0.551–0.85	Quartile 3 0.851–1.15	Quartile 4 > 1.15		
n	1257 (100)	74 (5.9)	493 (39.2)	492 (39.1)	198 (15.8)		
Positive TPOAb	206 (16.4)	22 (29.7)	74 (15.0)	76 (15.4)	34 (17.2)	10.703	0.013
Positive TGAb	166 (13.2)	21 (28.4)	56 (11.4)	53 (10.8)	36 (18.2)	23.148	0.000
HT^a^	140 (11.1)	20 (27.0)	43 (8.7)	55 (11.2)	22 (11.1)	21.785	0.000
Hypothyroidism^b^	161 (12.8)	30 (40.5)	49 (9.9)	55 (11.2)	27 (13.6)	54.527	0.000
Subclinical hypothyroidism	136 (10.8)	24 (32.4)	40 (8.1)	48 (9.8)	24 (12.1)	40.490	0.000
Clinical hypothyroidism	25 (2.0)	6 (8.1)	9 (1.8)	7 (1.4)	3 (1.5)	10.090	0.012
Hyperthyroidism^c^	19 (1.5)	0	6 (1.2)	10 (2.0)	3 (1.5)	4.008	0.216
Subclinical hyperthyroidism	6 (0.5)	0	1 (0.2)	3 (0.6)	2 (0.1)		
Clinical hyperthyroidism	13 (1.0)	0	5 (1.0)	7 (1.4)	1 (0.5)		

^a^HT: Hashimoto thyroiditis, which was diagnosed using ultrasonography; ^b^including clinical and subclinical hypothyroidism; ^c^including clinical and subclinical hyperthyroidism; TPOAb: anti-thyroid peroxidase antibody; TGAb: anti-thyroglobulin antibody.

After performing logistic regression analysis to adjust for confounding factors, the TGAb positivity rates in the lowest serum magnesium level (≤0.55 mmol/L) quartile group were higher than those in the quartile 3 group (0.851–1.15 mmol/L), (odds ratios [ORs]: 3.036–3.236); the prevalence of HT in the lowest serum magnesium level (≤0.55 mmol/L) quartile group was higher than that in the quartile 3 group (0.851–1.15 mmol/L), (ORs: 2.748–2.847); however, the serum magnesium level was not a statistically significant risk factor for TPOAb positivity (p > 0.05). The risks of hypothyroidism in the lowest serum magnesium level (≤0.55 mmol/L) quartile group were higher than those in the quartile 3 group (0.851–1.15 mmol/L), (ORs: 4.482–4.841); The risks of subclinical hypothyroidism in the lowest serum magnesium level (≤0.55 mmol/L) quartile group were higher than those in the quartile 3 group (0.851–1.15 mmol/L), (ORs: 4.517–4.971). The logistic regression analysis results are shown in Tables 3, 4 and 5. The results using the forward method, backward method, or all arguments analysed together were consistent.

**Table 3:** Relative risk of TPOAb and TGAb positivity according to quartiles of serum magnesium as determined using multiple logistic regression analyses.

Serum magnesium (mmol/L)	Model 1^a^		Model 2^b^		Model 3^c^		Model 4^d^	
	OR (95% CI)	*p*	OR (95% CI)	*p*	OR (95% CI)	*p*	OR (95% CI)	*p*
**Positive TPOAb**								
Serum magnesium		0.075		0.071		0.066		0.056
≤0.55	2.099 (1.162–3.792)	0.014	2.071 (1.146–3.744)	0.016	2.127 (1.167–3.874)	0.014	2.208 (1.222–3.990)	0.009
0.551–0.85	1.103 (0.759–1.605)	0.607	1.121 (0.770–1.633)	0.551	1.119 (0.764–1.638)	0.563	1.075 (0.740–1.563)	0.704
0.851–1.15	1.00		1.00		1.00		1.00	
>1.15	0.927 (0.586–1.468)	0.747	0.896 (0.565–1.422)	0.642	0.885 (0.552–1.417)	0.611	0.960 (0.607–1.519)	0.862
**Positive TGAb**								
Serum magnesium		0.002		0.003		0.002		0.001
≤0.55	3.084 (1.690–5.629)	0.000	3.036 (1.663–5.544)	0.000	3.236 (1.751–5.980)	0.000	3.171 (1.730–5.812)	0.000
0.551–0.85	1.186 (0.780–1.803)	0.425	1.190 (0.782–1.811)	0.417	1.211 (0.792–1.851)	0.377	1.158 (0.761–1.760)	0.494
0.851–1.15	1.00		1.00		1.00		1.00	
>1.15	1.586 (0.990–2.541)	0.055	1.557 (0.971–2.497)	0.066	1.484 (0.916–2.405)	0.109	1.484 (0.916–2.405)	0.043

TPOAb: anti-thyroid peroxidase antibody; TGAb: anti-thyroglobulin antibody; OR: odds ratio; CI: confidence interval.

^a^Model 1: adjusted for age, sex, smoking status, and serum iodine concentration;

^b^Model 2: additionally adjusted for body mass index;

^c^Model 3: adjusted for all covariates in model 2 as well as income and education;

^d^Model 4: adjusted for all covariates in model 1, but age was used as classification variable according to youth, middle age, and old age (as shown in Table 1).

Regression analyses using the forward method, backward method, and all arguments simultaneously were performed; the results were similar.

**Table 4:** Relative risk of Hashimoto thyroiditis (HT) diagnosed using ultrasonography according to quartiles of serum magnesium as determined using multiple logistic regression analyses.

Serum magnesium (mmol/L)	Model 1^a^		Model 2^b^		Model 3^c^		Model 4^d^	
	OR (95% CI)	*p*	OR (95% CI)	*p*	OR (95% CI)	*p*	OR (95% CI)	*p*
**HT using ultrasonography**								
Serum magnesium		0.002		0.002		0.003		0.001
≤0.55	2.748 (1.489–5.070)	0.001	2.847 (1.533–5.287)	0.001	2.763 (1.470–5.193)	0.002	2.944 (1.590–5.450)	0.001
0.551–0.85	0.884 (0.568–1.376)	0.585	0.916 (0.588–1.428)	0.700	0.900 (0.575–1.408)	0.644	0.871 (0.559–1.355)	0.539
0.851–1.15	1.00		1.00		1.00		1.00	
>1.15	0.806 (0.472–1.378)	0.430	0.785 (0.457–1.349)	0.381	0.755 (0.434–1.313)	0.320	0.832 (0.487–1.420)	0.499

HT: Hashimoto thyroiditis; OR: odds ratio; CI: confidence interval.

^a^Model 1: adjusted for age, sex, smoking status, and serum iodine concentration;

^b^Model 2: additionally adjusted for body mass index;

^c^Model 3: adjusted for all covariates in model 2 as well as income and education;

^d^Model 4: adjusted for all covariates in model 1, but age was used as a classification variable according to youth, middle age, and old age (as shown in Table 1).

Regression analyses using the forward method, backward method, and all arguments simultaneously were performed; the results were similar.

**Table 5:** Relative risk of hypothyroidism (including clinical and subclinical hypothyroidism) and subclinical hypothyroidism-only according to quartiles of serum magnesium determined using multiple logistic regression analyses.

	Model 1^a^		Model 2^b^		Model 3^c^		Model 4^d^	
	OR (95% CI)	*p*	OR (95% CI)	*p*	OR (95% CI)	*p*	OR (95% CI)	*p*
**Hypothyroidism**								
Serum magnesium		0.000		0.000		0.000		0.000
≤0.55	4.482 (2.438–8.239)	0.000	4.544 (2.468–8.369)	0.000	4.841 (2.584–9.070)	0.000	4.785 (2.582–8.866)	0.000
0.551–0.85	0.982 (0.627–1.535)	0.935	0.999 (0.636–1.570)	0.997	1.024 (0.647–1.621)	0.918	0.995 (0.634–1.559)	0.981
0.851–1.15	1.00		1.00		1.00		1.00	
>1.15	0.954 (0.561–1.623)	0.863	0.916 (0.536–1.565)	0.747	0.847 (0.491–1.461)	0.551	0.973 (0.572–1.657)	0.920
**Subclinical hypothyroidism**								
Serum magnesium		0.000		0.000		0.000		0.000
≤0.55	4.517 (2.382–8.567)	0.000	4.531 (2.382–8.617)	0.000	4.971 (2.557–9.666)	0.000	4.654 (2.435–8.896)	0.000
0.551–0.85	0.885 (0.540–1.451)	0.629	0.919 (0.558–1.512)	0.739	0.951 (0.572–1.580)	0.846	0.890 (0.543–1.461)	0.646
0.851–1.15	1.00		1.00		1.00		1.00	
>1.15	1.066 (0.608–1.872)	0.823	1.038 (0.589–1.831)	0.897	0.903 (0.505–1.613)	0.730	1.087 (0.619–1.910)	0.772

^a^Model 1: adjusted for age, sex, anti-thyroid peroxidase antibody, anti-thyroid globulin antibody, and serum iodine concentration.

^b^Model 2: additionally adjusted for smoking status, and body mass index.

^c^Model 3: adjusted for all covariates in model 2 as well as income and education.

^d^Model 4: adjusted for all covariates in model 1, but age was used as a classification variable according to youth, middle age, and old age (as shown in Table 1).

Regression analyses using the forward method, backward method, and all arguments simultaneously were performed and the results were similar.

OR: odds ratio; CI: confidence interval.

## Discussion

Magnesium in the human body is mostly located in the cells and bone tissues; only 1% of total body magnesium is located in extracellular fluids, and only 0.3% of total body magnesium is present in serum^1^. However, the detection of intracellular magnesium ions is difficult, while the detection of serum magnesium ions is simple and convenient. Therefore, serum magnesium is still used to assess individuals’ magnesium nutritional statuses^1^. Hypermagnesaemia and magnesium poisoning are rare in clinical practice, and only occur in patients with severe renal failure. The main clinical manifestation of magnesium nutrition imbalance is low serum magnesium, the definition of which varies among different populations and methods of detection. Recent evaluations of serum magnesium as an indicator of magnesium status have indicated that individuals with serum magnesium values >0.85 mmol/L most likely have adequate magnesium levels^20,28,29^. Therefore, in our study, serum magnesium levels ≤0.85 mmol/L were considered low. Additionally, serum magnesium levels ≤0.55 mmol/L were considered severely low in our study, as previously described in the literature^30,31^.

Moreover, the median serum magnesium level was 0.89 (0.73–1.06) mmol/L in our study; the proportion of subjects with inadequate magnesium levels (≤0.85 mmol/L) was 45.1%, while the proportion of people with severely low serum magnesium was 5.9%. The NAHSIT^4^ showed that the mean serum magnesium levels of male and female participants were 0.861 and 0.866 mmol/L, respectively; furthermore, 12.3% and 23.7% of male and female participants had low serum magnesium levels (defined as <0.8 mmol/L in the NAHSIT), which were lower rates than in our study. Using the Food Frequency Questionnaire, the NAHSIT found that the daily magnesium intakes of 75% in male participants and 81% in female participants were lower than the DRI; the daily magnesium intake of middle-aged individuals (ages 45–64 years) was the highest, while that of elderly individuals (≥65 years) was the lowest. Our study also found that the proportion of elderly subjects (≥65 years) was significantly higher in the lowest serum magnesium quartile group. Moreover, the proportion of young people (18–39 years) tended to decrease in the higher (>0.55 mmol/L) quartile groups, while the proportion of individuals ≥40 years old tended to increase. On the one hand, this might be related to the intake level of magnesium in the diet; on the other hand, these results can also be explained by the decline of intestinal absorption capacity in the elderly vs. rapid metabolism in young individuals. Our study also found that individuals with lower educational and income levels were more likely to exhibit low serum magnesium; this might be related to dietary structure as well as food affordability. In a cross-sectional study of 13,226 American participants, the proportion of individuals with serum magnesium levels <0.75 mmol/L was 26.5%, while that of individuals with levels <0.70 mmol/L was 10.3%. In that study, participants with lower education and income levels were more likely to have low serum magnesium levels^32^, which was consistent with our data.

Logistic regression analysis revealed that the morbidity risk owing to clinical and subclinical hypothyroidism was increased in the lowest serum magnesium level (≤0.55 mmol/L) quartile group. Early studies on magnesium and thyroid function revealed that serum magnesium levels in patients with hyperthyroidism are decreased while those in patients with hypothyroidism are increased; this change might be related to thyroid hormones causing increased magnesium excretion in the urine^33^. However, subsequent studies have produced contrasting findings; animal model^34^ and clinical studies^35,36^ found hypothyroidism to be associated with decreased serum magnesium levels, and the role of thyroid hormones on the magnesium urinary excretion rate was mainly related to their effect on the degree of urinary concentration. There were no differences in the levels of urinary magnesium or creatinine levels in patients with varying thyroid functionality^35^. Furthermore, a previous study showed that the inhibition of mitochondrial oxidative phosphorylation may lead to decreased iodine uptake by thyroid cells, as such uptake is achieved by a sodium iodide cotransporter that requires a mitochondrial energy supply^37^. Magnesium, as an enzyme cofactor, plays a critical role in mitochondrial oxidative phosphorylation and ATP synthesis, and its deficiency can affect these functions and lead to decreased iodine uptake by thyroid cells and a subsequent drop in thyroid hormone synthesis, thereby causing the secretion of thyroid-stimulating hormone (TSH). Animal experiments have shown that magnesium supplementation can significantly increase radioactive iodine uptake by thyroid cells, while its deficiency does the opposite^34^. Notably, the majority of participants with hypothyroidism in this study exhibited subclinical hypothyroidism; those with clinical hypothyroidism were too few in number to analyse separately using logistic regression analysis. Therefore, our conclusions are mainly applicable to subclinical hypothyroidism.

Our study found that serum magnesium levels ≤0.55 mmol/L were related to the risk of TGAb positivity and prevalence of HT. There are at least two explanations for this: first, severely low serum magnesium can increase TGAb via the abnormal activation of immune cells and induction of an autoimmune response. A study on patients with Graves’ disease found that their serum magnesium concentrations were lower than in normal individuals, and that the serum magnesium concentration was inversely related to the activation levels of CD3^+^, CD4^+^, CD8^+^T, and CD19^+^B cells. It was speculated that low serum magnesium might lead to decreased immune tolerance and abnormal activation of immune cells^26^. Second, given its function as a coenzyme, magnesium is involved in a variety of antioxidant metabolism pathways, such as glutathione synthesis; low serum magnesium could therefore reduce the antioxidant response capacity in cells and allow the accumulation of free radicals, resulting in oxidative stress and tissue damage^21,38,39^. Epidemiological studies have shown that insufficient magnesium intake is associated with a variety of chronic inflammatory diseases and elevated serum C-reactive protein levels^6,22,23,40^.

Our study revealed that severely low serum magnesium levels were not related to increased TPOAb positivity. The clinical significance of TPOAb is distinct from that of TGAb, as it is the most sensitive and specific index for the diagnosis of autoimmune thyroiditis and is closely related to hypothyroidism^41^. However, as a serological marker of autoimmune thyroiditis, TGAb does not damage the thyroid gland^42^. The different effects of severely low serum magnesium on the two autoantibodies examined in our study indicated that its effect on the thyroid autoantibody might primarily be caused by inflammation and oxidative stress, rather than by activating autoimmune responses. In other words, severely low serum magnesium is not the initiating factor of autoimmune thyroiditis, but might be an aggravating factor via inflammation.

This study found that non-severely low serum magnesium (0.551–0.85 mmol/L) is not associated with thyroid autoantibody levels or thyroid function. Another study on the relationship between low serum magnesium and metabolic syndrome with low-grade inflammation also found that inflammatory factors were elevated only when magnesium levels were severely low (<0.5 mmol/L)^31^. Animal studies found that the inflammatory response caused by mild-to-moderate magnesium deficiency could be compensated for, or aggravated, by other factors^39^. Hegsted *et al*. found that reducing the magnesium intake in rats to 50% of their required levels did not inhibit their growth; however, placing these rats in a cold environment (13 degrees) reduced their growth rate significantly compared with control rats with normal magnesium intake^43^. This indicated that the effects of mild versus moderate magnesium deficiency in different individuals might be more complex; therefore, studies of different populations may well produce different results. Additionally, results can differ based on different definitions of low serum magnesium levels and varying cut-off points.

This study included some limitations. First, because most of the body’s magnesium content is intracellular, serum magnesium does not fully represent the body’s magnesium nutritional status. However, in a large-scale epidemiological investigation, serum magnesium is still the most feasible and representative index. Second, dietary magnesium intake was not investigated in this study. If serum magnesium and the nutritional questionnaire were analysed together, more comprehensive data on the nutritional status of magnesium may have been obtained. Third, all subjects included in this study were residents of Tianjin; while the background data in each group were relatively consistent, other confounding factors might still be present. Lastly, as an observational study, our results could only reveal the associations between severely low serum magnesium levels, TGAb, HT, and thyroid function; prospective interventional studies are required to confirm the conclusion and reveal any cause-and-effect relationships.

## Conclusions

Our cross-sectional survey revealed that the proportion of Tianjin residents with inadequate magnesium status (serum magnesium levels ≤0.85 mmol/L) was 28.2%, and that with severe magnesium deficiency (serum magnesium levels ≤0.55 mmol/L) was 5.9%. Serum magnesium levels ≤0.55 mmol/L were associated with increased risks of TGAb positivity, the prevalence of HT, and (mainly subclinical) hypothyroidism, indicating that serum magnesium levels should be evaluated in patients with autoimmune thyroiditis and hypothyroidism. Increased magnesium intake or magnesium supplementation may be beneficial for patients with severely low blood magnesium who are diagnosed with these disorders.

## Methods

### Subjects

The “Thyroid Disorders, Iodine Status and Diabetes: a National Epidemiological Survey-2014” is a cross-sectional study of thyroid disease, iodine nutrition status, and diabetes mellitus performed across 31 provinces, municipalities, and autonomous regions of China; the Tianjin portion was conducted between June and September, 2015. A total of 2,650 participants were enrolled using stratified multi-stage cluster random sampling. The serum magnesium levels of 1,257 participants whose blood samples were kept intact were examined in this study; all participants were older than 18 years and were of Han ethnicity. All participants had lived in the local area for more than five years; none received any examination involving iodinated contrast agent or took any drugs containing iodine during the previous three months. Pregnant women and patients with chronic diarrhoea and kidney disease were excluded. The research project was approved by the ethics committee of The First Hospital of China Medical University and was conducted in accordance with the Declaration of Helsinki II. All participants signed informed consent forms.

### Specimen and data acquisition

Demographic data, smoking history (including passive smoking), and income and education levels were collected via questionnaires. The heights and weights of all participants were measured by a single investigator using a standardized measurement method, and the BMI was then calculated. Fasting venous blood (5 mL) was collected from all participants in the morning, and blood was allowed to coagulate. Serum was separated within 6 h and stored in a −80 °C freezer; the levels of TSH, TPOAb, TGAb, serum iodine, and serum magnesium were detected uniformly. Free thyroxine (FT_4_) was also detected for participants with TSH elevation; FT_4_ and free triiodothyronine (FT_3_) were also detected in participants with decreased TSH levels. Fasting morning urine (5 mL) was collected from all participants and stored at −80 °C for urinary iodine tests.

### Laboratory testing

Roche cobas e601 reagent kits were used for the detection of FT_3_, FT_4_, TSH, TPOAb, and TGAb; all were detected using chemiluminescence immunoassays according to the manufacturer’s instructions. The kits were subjected to quality control tests; the intra-assay coefficients of variation for the measured parameters were 1.1–6.3%, while the inter-assay coefficients of variation were 1.9–9.5%. Serum iodine and serum magnesium were detected using inductively coupled plasma mass spectrometry. The UIC was detected by urine iodine arsenic cerium catalytic spectrophotometry (WS/T-2006). The levels of serum iodine, serum magnesium, and UIC were the averages of triplicate measurements. The intra-assay coefficients of variation for serum iodine and serum magnesium were 1.4–4.5%, while the inter-assay coefficients of variation were 2.7–6.4%. The intra-assay coefficient of variation for UIC (66 μg/L) was 3–4%, while the inter-assay coefficients of variation were 4–6%; the intra-assay coefficients of variation for UIC (230 μg/L) were 2–5%, while the inter-assay coefficients of variation were 3–6%.

### Ultrasonography

Thyroid ultrasonography was performed by the same experienced physician using commercially available ultrasound equipment (LOGIQα100, GE Company, United States) equipped with a 7.5 Hz linear transducer. Patients were examined in a supine position with their neck hyperextended in accordance with a standard sonographic protocol. Markedly decreased echogenicity, heterogeneity, and fibrous septation infiltration were considered indicative of HT according to the literature^44,45^.

### Diagnostic criteria

Using the normal ranges provided by the testing kits as references, the normal range of TSH was 0.27–4.20 mIU/L, while the reference range of FT_4_ was 12.00–22.00 pmol/L. Patients with TSH elevation and decreased FT_4_ combined with positive thyroid autoantibodies or ultrasound performance of HT were deemed to have clinical hypothyroidism; those with TSH elevation and normal FT_4_ were considered to have subclinical hypothyroidism. The reference ranges for TPOAb and TGAb were 0–34 IU/L and 0–115 IU/L, respectively. TPOAb >34 IU/L and TGAb >115 IU/L were deemed to be elevated (i.e., positive).

### Statistical analysis

The SPSS 19.0 software (IBM, Chicago, Illinois) was used to analyse the data. The measurements and normal distributions are expressed as means ± standard deviations, while values with skewed distributions are expressed as medians (interquartile ranges); enumerated values are represented as percentages. The participants were divided into four groups based on serum magnesium concentration: quartile 1, serum magnesium concentration ≤0.55 mmol/L; quartile 2, 0.551–0.85 mmol/L; quartile 3, 0.851–1.15 mmol/L; and quartile 4, >1.15 mmol/L corresponding to severe magnesium deficiency, deficiency, adequate level, and excess, respectively. The adequate magnesium group (0.851–1.15 mmol/L) was taken as the control group. Sex, TPOAb, TGAb, smoking history, annual income level, education level, and BMI were used as classification variables; distributions are shown in Table 1. Age was used as both a continuous and classification variable. BMI categories were determined according to the overweight and obesity standards in China^46^. The data in each group were analysed using single factor analysis of variance and rank sum tests; the chi-square and Fisher exact probability tests were used to compare the rates among groups.

A logistic regression model was used to adjust for the influence of confounding factors. When analysing the dependent variables TPOAb, TGAb, and HT, the independent variables sex, age, serum iodine concentration, serum magnesium concentration, and smoking history were incorporated into model 1. Model 2 incorporated model 1 and additionally adjusted for BMI; furthermore, model 3 incorporated model 2 while adding the independent variables of income and education levels. When analysing the dependent variables (all hypothyroidism and subclinical hypothyroidism-only), the independent variables sex, age, TPOAb, TGAb, serum iodine concentration, and serum magnesium concentration were incorporated into model 1. Model 2 incorporated model 1 plus the independent variables smoking history and BMI, while model 3 incorporated model 2 in addition to the independent variables of income and education levels. Age and serum iodine concentration were continuous variables, whereas the remaining variables were categorical. The forward and backward methods, as well as all arguments analysed simultaneously, were used for regression analysis; p < 0.05 was considered statistically significant.

### Availability of data and materials

The datasets analysed during the current study are available from the corresponding author on reasonable request.

### Ethics Approval and Consent to Participate

Ethical approval for this study was obtained from the Ethics Committee of The First Hospital of China Medical University, Shenyang, China. Informed consent was obtained from all participants.

## References

[1] Gröber U, Schmidt J, Kisters K (2015). Magnesium in prevention and therapy. Nutrients..

[2] Volpe SL (2013). Magnesium in disease prevention and overall health. Adv. Nutr..

[3] Moore-Schiltz L, Albert JM, Singer ME, Swain J, Nock NL (2015). Dietary intake of calcium and magnesium and the metabolic syndrome in the National Health and Nutrition Examination (NHANES) 2001–2010 data. Br. J. Nutr..

[4] Wang JL, Weng YL, Pan WH, Kao MD (2011). Trends and nutritional status for magnesium in Taiwan from NAHSIT 1993 to 2008. Asia Pac. J. Clin. Nutr..

[5] Ayuk J, Gittoes NJ (2014). Contemporary view of the clinical relevance of magnesium homeostasis. Ann. Clin. Biochem..

[6] Kim DJ (2010). Magnesium intake in relation to systemic inflammation, insulin resistance, and the incidence of diabetes. Diabetes Care..

[7] Fang X (2016). Dietary magnesium intake and the risk of cardiovascular disease, type 2 diabetes, and all-cause mortality: a dose-response meta-analysis of prospective cohort studies. BMC Med..

[8] He K (2006). Magnesium intake and incidence of metabolic syndrome among young adults. Circulation..

[9] Kass L, Weekes J, Carpenter L (2012). Effect of magnesium supplementation on blood pressure: a meta-analysis. Eur. J. Clin. Nutr..

[10] Shlezinger M (2016). Desalinated seawater supply and all-cause mortality in hospitalized acute myocardial infarction patients from the Acute Coronary Syndrome Israeli Survey 2002–2013. Int. J. Cardiol..

[11] Akarolo-Anthony SN (2014). Plasma magnesium and risk of ischemic stroke among women. Stroke..

[12] Gaul C, Diener HC, Danesch U, Migravent® Study Group (2015). Improvement of migraine symptoms with a proprietary supplement containing riboflavin, magnesium and Q10: a randomized, placebo-controlled, double-blind, multicenter trial. J. Headache Pain..

[13] Nogovitsina OR, Levitina EV (2006). Effect of MAGNE-B6 on the clinical and biochemical manifestations of the syndrome of attention deficit and hyperactivity in children. Eksp. Klin. Farmakol..

[14] Barbagallo M (2011). Altered ionized magnesium levels in mild-to-moderate Alzheimer’s disease. J. Magnes. Res..

[15] Skobeloff EM, Spivey WH, McNamara RM, Greenspon L (1989). Intravenous magnesium sulfate for the treatment of acute asthma in the emergency department. JAMA..

[16] Li FY (2011). Second messenger role for Mg2+ revealed by human T-cell immunodeficiency. Nature..

[17] Deason-Towne F, Perraud AL, Schmitz C (2012). Identification of Ser/Thr phosphorylation sites in the C2-domain of phospholipase Cgamma2 (PLCgamma2) using TRPM7-kinase. J. Cell Signal..

[18] Sahni J (2010). TRPM7 regulates quiescent/proliferative metabolic transitions in lymphocytes. Cell Cycle..

[19] Kuras Z (2012). KCa3.1 and TRPM7 channels at the uropod regulate migration of activated human T cells. PLoS One..

[20] Nielsen FH (2018). Magnesium deficiency and increased inflammation: current perspectives. J. Inflamm. Res..

[21] Yamanaka R (2016). Mitochondrial Mg(2+) homeostasis decides cellular energy metabolism and vulnerability to stress. Sci. Rep..

[22] Chacko SA (2010). Relations of dietary magnesium intake to biomarkers of inflammation and endothelial dysfunction in an ethnically diverse cohort of postmenopausal women. Diabetes Care..

[23] Chacko SA (2011). Magnesium supplementation, metabolic and inflammatory markers, and global genomic and proteomic profiling: a randomized, double-blind, controlled, crossover trial in overweight individuals. Am. J. Clin. Nutr..

[24] Contempré B (1992). Effect of selenium supplementation on thyroid hormone metabolism in an iodine and selenium deficient population. Clin. Endocrinol. (Oxf)..

[25] Hess SY, Zimmermann MB, Adou P, Torresani T, Hurrell RF (2002). Treatment of iron deficiency in goitrous children improves the efficacy of iodized salt in Cote d’Ivoire. Am. J. Clin. Nutr..

[26] Klatka M, Grywalska E, Partyka M, Charytanowicz M, Rolinski J (2013). Impact of methimazole treatment on magnesium concentration and lymphocytes activation in adolescents with Graves’ disease. Biol. Trace Elem. Res..

[27] Moncayo R, Moncayo H (2014). The WOMED model of benign thyroid disease: Acquired magnesium deficiency due to physical and psychological stressors relates to dysfunction of oxidative phosphorylation. BBA Clin..

[28] Nielsen FH, Johnson LA (2017). Data from Controlled Metabolic Ward Studies Provide Guidance for the Determination of Status Indicators and Dietary Requirements for Magnesium. J. Biol. Trace Elem. Res..

[29] Costello RB (2016). Perspective: The Case for an Evidence-Based Reference Interval for Serum Magnesium: The Time Has Come. J. Adv. Nutr..

[30] Lutsey PL (2014). Serum magnesium, phosphorus, and calcium are associated with risk of incident heart failure: the Atherosclerosis Risk in Communities (ARIC) Study. Am. J. Clin. Nutr..

[31] Guerrero-Romero F, Bermudez-Peña C, Rodríguez-Morán M (2011). Severe hypomagnesemia and low-grade inflammation in metabolic syndrome. Magnes. Res..

[32] Tin A (2015). Results from the Atherosclerosis Risk in Communities study suggest that low serum magnesium is associated with incident kidney disease. Kidney Int..

[33] Jones JE, Desper PC, Shane SR, Flink EB (1966). Magnesium metabolism in hyperthyroidism and hypothyroidism. J. Clin. Invest..

[34] Humphray HP, Heaton FW (1972). Relationship between the thyroid hormone and mineral metabolism in the rat. J. Endocrinol..

[35] Dolev E (1988). Alterations in magnesium and zinc metabolism in thyroid disease. Metabolism..

[36] Al-Hakeim HK (2009). Serum levels of lipids, calcium and magnesium in women with hypothyroidism and cardiovascular diseases. J. Lab. Physicians..

[37] Tyler DD, Gonze J, Lamy F, Dumont JE (1968). Influence of mitochondrial inhibitors on the respiration and energy-dependent uptake of iodide by thyroid slices. Biochem. J..

[38] Morais JB (2017). Role of magnesium in oxidative stress in individuals witho. Biol Trace Elem. Res..

[39] Nielsen FH (2010). Magnesium, inflammation, and obesity in chronic disease. Nutr. Rev..

[40] de Oliveira AR (2015). Magnesium status and its relationship with C-reactive protein in obese women. Biol. Trace Elem. Res..

[41] Kotani T (1998). Anti-TPO autoantibodies. Rinsho Byori..

[42] Hollowell JG (2002). Serum TSH, T(4), and thyroid antibodies in the United States population (1988 to 1994): National Health and Nutrition Examination Survey (NHANES III). J. Clin. Endocrinol. Metab..

[43] Kramer JH, Mak IT, Phillips TM, Weglicki WB (2003). Dietary magnesium intake influences circulating pro-inflammatory neuropeptide levels and loss of myocardial tolerance to postischemic stress. Exp. Biol. Med. (Maywood)..

[44] Willms A (2013). Correlation between sonography and antibody activity in patients with Hashimoto thyroiditis. J. Ultrasound Med..

[45] Ceylan I (2014). Roles of ultrasound and power Doppler ultrasound for diagnosis of Hashimoto thyroiditis in anti-thyroid marker-positive euthyroid subjects. Quant. Imaging Med. Surg..

[46] Endocrinology Association Obese Group of Chinese Medical Association (2011). Expert consensus of prevention and treatment on Chinese adult obesity. Chin. J. Endocrinol. Metab..

